# **Universal Health Coverage for Non-communicable Diseases and Health Equity: Reflections on the Role of Ideas and Democratic Decision-Making** Comment on "Universal Health Coverage for Non-Communicable Diseases and Health Equity: Lessons from Australian Primary Healthcare"

**DOI:** 10.34172/ijhpm.2021.117

**Published:** 2021-08-30

**Authors:** Lauren Paremoer

**Affiliations:** Department of Political Studies, University of Cape Town, Cape Town, South Africa.

**Keywords:** Social Determinants of Health, Universal Health Coverage, Health Equity, Health Governance

## Abstract

Fisher et al have published a thought-provoking article exploring the complex relationship between universal health coverage (UHC) and equity. This commentary builds on two of the lessons they highlight: the importance of ideas in determining how exactly UHC advances equity, and the political difficulties of addressing the commercial determinants of health. I argue that equity in UHC can be advanced through interventions that address popular prejudices against public health systems, greater emphasis on structural and commercial drivers of ill-health in health professionals’ training, and by ensuring meaningful public participation in decision-making about the institutionalisation and management of UHC. These strategies are important for ensuring that the political, power-laden nature of concepts such as "universality", "health" and "care" are explicitly acknowledged and publicly debated – rather than continuing the current trend of allowing technocrats to reduce UHC to a matter of efficiently and expeditiously financing curative healthcare services.

## Background

 Fisher et al have published a thought-provoking article that explores the complex relationship between universal health coverage (UHC) and equity, particularly equitable access to care for non-communicable diseases (NCDs). The authors use an analytical framework that identifies the interplay of ideas, actors and structures that have shaped Australians’ access to primary healthcare for NCDs between 2008 and 2018. In this commentary, I retain their definition of UHC (“a health financing scheme to enable people to access healthcare – especially primary healthcare – without suffering financial hardship”)^1^ and comprehensive primary healthcare (CPHC) (“comprehensive first-level care that incorporates but extends beyond primary medical care to include health promotion, disease prevention, community engagement and action to address SDH [social determinants of health]; and public regulation of key social determinants of NCDs, such as the products and practices of tobacco, food and alcohol industries”).^1^

 Significantly, this definition of UHC defines it as both a financing mechanism and a regulatory regime. That is, UHC financing mechanisms should ensure affordability of, and equitable access to health services. Additionally, this financing regime should be supplemented by regulation of the commercial determinants that drive the emergence of conditions like NCDs which add to demand for healthcare services (and public financing for those services).

 One of the paper’s major strengths is that it acknowledges that UHC may decrease the financial barriers to accessing healthcare but does not conflate this with universal and equitable access to CPHC for people living with NCDs. It shows that effective management of NCDs is significantly undermined when UHC is delivered through financing mechanisms that prioritise episodic rather than continuous care (eg, where fee-for-service reimbursements to general practitioners (GPs) is the dominant mode of financing, together with weak investment in allied health services), or fail to incentivise greater provision of care in underserved “regional, rural or remote” areas.^1^

 In what follows I expand on two findings mentioned in the paper: the importance of ideas in determining in which respects UHC advances equity, and the difficulties of addressing the commercial determinants of health, ie, the “strategies and approaches used by the private sector to promote products and choices that are detrimental to health.”^2^ I develop these aspects of the paper with a view to highlighting their importance in securing a just and equitable infrastructure for UHC in countries where UHC is still in the early stages of institutionalisation.

###  The Importance of Ideas 

 The authors identify three ideas that support equitable access to CPHC services for NCDs in UHC systems: embracing broad psychosocial models of health, prioritising preventative care and need-based access to care, and improving the SDHs in parallel with reducing the financial costs of healthcare. These ideas shape policy-makers’ thinking in countries where UHC is already a reality.

 Alongside this it is also necessary to acknowledge the importance of “common sense” or hegemonic ideas about the desirability of UHC, particularly in countries with mixed health systems that aim to use UHC to strengthen the public health system and to regulate the commercial determinants of health. In countries where the public healthcare system has historically been neglected by the government, particularly its CPHC dimension, public facilities have been stigmatised as largely dysfunctional and as service providers of last resort - particularly by the elites and middle classes, but also by the poor and precariously positioned, who typically don’t have the financial resources to opt out of public healthcare services that they sometimes experience as unreliable, disrespectful and sub-standard.^3^ These perceptions may play a significant role in preserving the role of the private sector in delivering UHC, especially if governments cannot convince prospective users of UHC-financed public sector services that UHC will prioritise and deliver tangible improvements in physical infrastructure, access to essential medicines, and timely care in the public sector.

 In short, Fisher et al perhaps fail to acknowledge how sometimes justified scepticism of public healthcare might generate political support for exactly the kind of inequitable UHC financing and service provision models this paper warns against: the retention of private health insurance as a component of UHC, state-funded financing for GP-centred episodic care rather than community health clinics comprised of multi-disciplinary teams, absence of healthcare services in rural areas, and a broader commitment to continued commercialisation of health services, ie, “the process of applying market principles to the functioning of policies and systems.”^4^Such a model may well lower financial barriers to accessing care but its efforts to preserve “consumer choice” limits financial support for publicly provided, comprehensive and preventative healthcare interventions.

###  Health Professionals’ Ideas 

 Fisher et al show that the vested interests of for-profit actors such as medical specialists, GPs, and private corporations shape UHC systems over time: their influence complicates the introduction of financing mechanisms and regulations that divert funding to public healthcare (as opposed to private care or private medical insurance schemes), or reduce the authority of independent GPs as the primary decision-makers or the default entry point into the health system (by instead positioning multi-disciplinary primary healthcare teams in this manner).

 In many low- and middle-income countries with mixed health systems that seek to institutionalise UHC models, GPs make up an influential constituency because they can offer services that supplement those offered by an over-stretched public health system, they contribute to UHC financing through taxes, and their interests often align with powerful players such as private medical insurance schemes. Where private GPs are retained as service providers under UHC schemes, their commercial interests might therefore play an important role in shaping emerging definitions of “quality” healthcare in favour of curative rather than preventative care. Health professionals’ training should include a critical engagement with the political economy of health, their location within it and the tensions these dynamics generate with respect to their ethical and professional obligation to “first do no harm.”

 A significant body of research suggests that phenomena such as racism and class status have historically played a significant role in shaping healthcare systems and medical knowledge, and continue to impact health outcomes, even in UHC systems.^5^ These systems of domination inevitably shape medical professionals’ ideas about who is most deserving of their care, and are important in modulating the extent to which need, equity and prevention feature in their engagements with patients – particularly patients from social groups that are stigmatised and marginalised (eg, undocumented people, sex workers, or racial minorities).

 This has implications for the training of health workers, whose academic and practical training should function as an important counterweight to structural incentives to pursue biomedical and behaviouralist modes of care that ignore the importance of the social and commercial determinants of health. In this regard, policy-making can make a difference by institutionalising policies that expose and counteract structural racism and discrimination against marginalised and stigmatised groups. Given the global rise of xenophobia and nativist rhetoric in recent years, it is particularly important to conscientise medical professionals about the ways in which their engagements with patients are enmeshed and potentially informed by larger racist, xenophobic and classist ideas aimed at maintaining social distance between stigmatised and dominant social groups.

 Such an orientation to training medical professionals is more likely to increase the efficacy of another suggestion put forward by Fisher et al, ie, that equity in UHC should be promoted through “regulatory measures and incentives to ensure a distribution of services and personnel that matches community needs in different areas.” At the level of the population or community, such measures can promote greater geographic proximity to care. However, at the level of doctor-patient interaction, caregivers’ perceptions of the validity of patients’ accounts of their needs, symptoms, ability to comply with medical prescriptions without structured support, and demands for culturally appropriate care are conditioned by their awareness of the ways in which patients are dehumanised by broader structural processes.

###  Popular Governance in Public Health 

 UHC is shaped by the equity-impeding effects of the commercial determinants of health.^1^ As such, UHC promotes better access to quality CPHC, as well as prevention and care for NCDs, provided that it is shaped by “[p]olicy decision-making processes that limit the influence of sectional groups with financial interests in policy settings, including medical professionals, PHI, and the tobacco, food and alcohol industry sectors.”^1^ In terms of NCD prevention, Fisher et al argue for regulations that “limit [the] impacts of corporatized food, alcohol and tobacco sectors on NCDs.”^1^ The political power of these industries, ie, their ability to make decisions about resource allocation and the norms that govern the life (and health) of the political community, functions as a distal, structural driver of the poor health outcomes that UHC aims to manage.

 Research shows that the political influence of for-profit entities like the food, alcohol, tobacco, and for-profit health industries create a regulatory environment in which people are more prone to developing NCDs and in which they find it more difficult to manage these conditions.^6^ The authors therefore recommend the creation of policy-making processes that limit the influence of these industries. I would argue that it is necessary to go beyond this, and to consider how one might build policy-making processes that expand the influence of groups that have been marginalised in health governance. For example, the article points out that equitable CPHC for NCDs is promoted by regional organisations or health authorities that have the resources and legal authority “to undertake population health planning and workforce planning, ensure coordination between primary, secondary and tertiary care services, and broker inter-sectoral partnerships to address SDH.”^1^ Such institutions could be sites for building public competencies and political influence in the decision-making processes about how UHC is organised and governed at the regional level, and forums for building “voice” and articulating demands about desired policy-changes at national level – including some of the policy changes recommended in this paper.

 This issue of popular political engagement in health governance is particularly important in light of the authors’ claim that “[e]quitable UHC is most likely to be achieved in publicly funded and managed health systems.”^1^ If current and prospective users of the public health system benefit from meaningful engagement in decision-making processes aimed at improving public healthcare, perhaps this would build the political support, class coalitions, and trust in public health institutions required to move towards greater investment in public health systems and more stringent regulation of the tobacco, food, and alcohol industries.

 There are no guarantees that such reforms of health governance will deliver this result: absent sufficient organising power at the grassroots level such reforms could result in interest groups or individuals who are largely disconnected from social movements and community organisations capturing these processes to advance their own interests. Another possibility is that inclusion in these processes could be largely symbolic, or that the public becomes disillusioned by perceived corruption or inefficiencies within the public health sector (thereby making them more amenable to supporting commercialised modes of realising UHC), or that citizens try and prevent non-citizens from accessing “new and improved” public health services.

 In practice these questions cannot be settled a priori. However, if limiting the scope of “sectional groups with financial interests in policy settings”^1^ is vital to producing more equitable universal health systems,^7^ investing in institutions that develop popular counter-power to such interests is a vital “social vaccine”^8^ against the commercialisation of healthcare. More fundamentally, I would argue, investing in popular governance of health is also a necessary strategy for ensuring that the political, power-laden nature of concepts such as “universality,” “health” and “care” are explicitly acknowledged and publicly debated – rather than continuing the current trend of giving technocrats the authority to reduce UHC to a matter of efficiently and expeditiously financing curative healthcare services to address the biomedical excesses of unhealthy societies.^9^

## Acknowledgements

 The author would like to thank the reviewers for their insightful and helpful comments on the first draft of this commentary.

## Ethical issues

 Not applicable.

## Competing interests

 Author declares that she has no competing interests.

## Author’s contribution

 LP is the single author of the paper.
